# A Critical Evaluation of the Effect of Electrode Thickness and Side Reactions on Electrolytes for Aluminum–Sulfur Batteries

**DOI:** 10.1002/cssc.202000447

**Published:** 2020-05-15

**Authors:** John Lampkin, He Li, Liam Furness, Rinaldo Raccichini, Nuria Garcia‐Araez

**Affiliations:** ^1^ Department of Chemistry University of Southampton University Road Southampton SO17 1BJ United Kingdom; ^2^ Current address: National Physical Laboratory Hampton Road Teddington Middlesex TW11 0LW United Kingdom

**Keywords:** batteries, electrochemistry, electrolytes, energy storage, reaction mechanisms

## Abstract

The high abundance and low cost of aluminum and sulfur make the Al–S battery an attractive combination. However, significant improvements in performance are required, and increasing the thickness and sulfur content of the sulfur electrodes is critical for the development of batteries with competitive specific energies. This work concerns the development of sulfur electrodes with the highest sulfur content (60 wt %) reported to date for an Al–S battery system and a systematic study of the effect of the sulfur electrode thickness on battery performance. If low‐cost electrolytes made from acetamide or urea are used, slow mass transport of the electrolyte species is identified as the main cause of the poor sulfur utilization when the electrode thickness is decreased, whereas complete sulfur utilization is achieved with a less viscous ionic liquid. In addition, the analysis of very thin electrodes reveals the occurrence of degradation reactions in the low‐cost electrolytes. The new analysis method is ideal for evaluating the stability and mass transport limitations of novel electrolytes for Al–S batteries.

## Graphical abstract

**Thick and thicker**! The thickness of the cathode and viscosity of the electrolyte affect the rate of transport of electrolyte species inside the porous sulfur electrode in Al–S batteries and cause poor sulfur utilization in deep eutectic solvents, whereas complete sulfur utilization is achieved with a less viscous ionic liquid. In addition, the analysis of the effect of cathode thickness on battery performance and the electrochemistry of carbon additives provides evidence of the degradation of deep eutectic solvents in Al–S batteries.

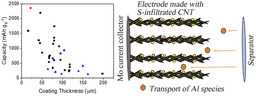

## Introduction

Lithium‐ion batteries are currently the best performing rechargeable batteries on the market and are used in mobile devices, electric vehicles and various other small‐scale energy storage systems. As the demand for cheaper batteries with larger energy capacities increases, researchers are turning towards alternatives and developing new battery systems. Many factors have contributed to this, including the limited lithium natural resources,^1^ the flammable nature of organic‐based solvents,^2^ the limited practical specific energy achievable by lithium‐ion batteries, and difficulty in recycling.^3^

A variety of battery technologies will be required to meet the energy storage needs in coming years and the aluminum–sulfur battery is likely to play an important role, because it is made of cheap and highly abundant elements and it can potentially achieve very high values of energy per unit mass or volume. Aluminum is highly abundant and as an anode material has very high theoretical values of gravimetric and volumetric capacity (2980 mAh g^−1^ and 8040 mAh cm^−3^ respectively).^4^ Coupling an aluminum anode with a sulfur cathode, another abundant material, produces a battery with a theoretical voltage of approximately 1.23 V, high theoretical specific energy (1319 Wh kg^−1^), and high theoretical energy density (2981 Wh L^−1^).^5^

A summary of reported studies on the development of Al–S batteries to date is provided in Table 1. It is clear that the Al–S battery is in its infancy, but promising high values of specific capacity have been achieved. These early studies demonstrate the potential of Al–S batteries as an alternative low‐cost battery, but much more work is required to improve performance to pave the way towards commercialization.^5^ To our knowledge, all previous studies (Table 1) have been performed with very low loadings of sulfur in the cathode (which is defined as the mass of sulfur per geometrical area of the cathode). In addition, the sulfur content in the cathode (% of the total mass of the cathode coating that is sulfur) is also rather low. However, sulfur electrodes with high sulfur loading and high sulfur content are required to meet the requirements of commercial applications of high specific energy and high energy density. To illustrate this point, the calculation of the specific energy of the cells normalized by the total mass of the sulfur and aluminum electrodes is included in Table 1. Although these values are still promising, further decreases in specific energy will occur owing to the mass of other passive components (e.g., electrolyte, current collectors), but the effect of the latter will be minimized by increasing the mass fraction of the active materials in the battery by increasing the sulfur loading in the sulfur electrode.^6^

**Table 1:** Comparison of the main electrochemical results reported in Al–S battery studies, in chronological order (from oldest to newest).

Ref.	Electrode composition	Electrolyte	Sulfur content [wt %]	Sulfur loading [mg cm^−2^]	Specific current [mA g_s_^−1^]	Initial specific discharge capacity [mAh g_S_^−1^]	Discharge voltage^[a]^ [V]	Specific energy of initial discharge^[b]^ [Wh kg_electrodes_^−1^]
8	S, Ketjen black, PVDF (50:30:20) on stainless steel (non‐rechargeable)	EMICl‐AlCl_3_ (1:1.5)	50	1.1	30	1400	1.2	656
4a	S on activated carbon cloth	EMICl‐AlCl_3_ (1:1.3)	n/a	0.8–1.0	50	1320	0.65	n/a
9	spreading the mixture of S and ionic liquid electrolyte onto activated CNF paper (S/CNF≈1:2)	EMICl‐AlCl_3_ (1:1.3)	33	≈1.0	≈30 (C/50)	1350	1.05	395
10	spreading the mixture of S and ionic liquid electrolyte onto activated CNF paper (S/CNF≈1:2)	0.5 m LiCF_3_SO_3_ in EMICl‐AlCl_3_ (1:1.25)	33	≈1.0	≈30 (C/50)	1250	0.76	265
11	S, CMK‐3, Ketjen black, PTFE (40:40:10:10)	EMIBr‐AlCl_3_ (1:1.3)	40	n/a	251	1500	≈0.5	245
		NBMPBr‐AlCl3 (1:1.3)	40	n/a	251	1390	≈0.5	227
12	S, MWCNT, polyacrylic latex (10:80:10) on Ni foil	urea‐AlCl_3_ (1:1.4)	10	0.42	1000	740	≈1.7	119
13	10 % S. SPAN, Ketjen black and PTFE (80:10:10) on carbon paper.	EMICl‐AlCl_3_ (1:1.5)	10	0.12	25	320	0.3	9
14	S, CMK‐3 1:1, with 10 % PTFE and 10 % Super C on Mo foil	acetamide‐AlCl_3_ (1:1.3)	40	0.25	100	2100	0.55	377
15	BN/S/C (6:1:2) with 10 % PVDF coated on Pt coated OHP organic film	EMICl‐AlCl_3_ (1:1.3)	10	≈0.3	100	≈275	1.15	≈30
this work	S, CNT with PEO and PVP coated on Mo foil (58.8:29.4:7.9:3.9)	EMICl‐AlCl_3_ (1:1.5)	58.8	≈0.4–3.5	50	1404^[c]^	0.31	192
		acetamide‐AlCl_3_ (1:1.5)	58.8	≈0.4–3.5	50	2129^[c]^	0.42	395
		urea‐AlCl_3_ (1:1.5)	58.8	≈0.4–3.5	50	2359^[c]^	0.41	428

[a] The discharge voltage from this work is the average voltage calculated by integrating the discharge voltage over the discharge capacity and then dividing by the total discharge capacity. The calculation procedure can be found in the Supporting Information (Equation S1). Because the average voltage has not been reported in previous published papers, the discharge voltage plateau is reported instead. [b] The specific energy of the initial discharge is normalized to the total mass of the sulfur and aluminum electrodes, and the calculation procedure can be found in the Supporting Information (Equation S2). [c] The thinnest mass loading (≈0.4 mg cm^−2^) is used when reporting the specific capacity and specific energy.

This work presents a systematic study of the effect of sulfur loading on the performance of Al–S batteries, which enables the identification of the critical issues that need to be addressed for the development of Al–S batteries to meet the requirements towards commercialization. The measurements were performed with advanced cathode formulations containing a relatively high sulfur content (≈60 wt %), which is the highest reported value for the Al–S battery system. The cathodes were prepared by doctor‐blade coating a thick slurry on an inert Mo foil, because Mo is a stable material in Al‐ion electrolytes.^7^ All the materials employed in the electrochemical cells were carefully selected to be resistant against degradation in contact with the corrosive Al‐ion electrolytes. Three different types of electrolytes were selected for this study, which provides new, unprecedented understanding into the reaction mechanism and differences in chemical stability of the different electrolytes in Al–S battery reactions.

The first electrolyte selected for this study is the most commonly used electrolyte in Al–S batteries: the ionic liquid 1‐ethyl‐3‐methylimidazolium chloride (EMIMCl)–AlCl_3_, which is made by mixing AlCl_3_ with the organic cation chloride salt EMIMCl. AlCl_3_ is a Lewis acid that is added in excess to facilitate the reversible electrochemical process of Al stripping/plating;^16^ here, an EMIMCl/AlCl_3_ molar ratio of 1:1.5 is used, which results in an electrolyte containing equimolar concentrations of AlCl_4_^−^ and Al_2_Cl_7_^−^.^17^

The other two electrolytes used were deep eutectic solvents made by mixing a Lewis base with AlCl_3_, in which AlCl_3_ is added in excess, again to facilitate reversible Al electrochemistry. Deep eutectic solvents have a significant cost advantage over ionic liquids while the conductivity and suitability for Al plating/stripping reactions is maintained.^18^ Here, we used either acetamide or urea as the Lewis base, and the resulting electrolytes are called acetalumina (acetamide/AlCl_3_ in a 1:1.5 molar ratio) and uralumina (urea/AlCl_3_ in a 1:1.5 molar ratio). The actual species present in the deep eutectic solvents are thought to be a mixture of aluminum species complexed with chloride anions and acetamide/urea ligands.^19^

The evaluation of the Al–S battery electrochemical performance as a function of sulfur loading presented here demonstrates that, unfortunately, the chemical stability of the chosen deep eutectic solvents is currently insufficient for Al–S battery applications, whereas the ionic liquid EMIMCl–AlCl_3_ appears to be much more stable. Further work is required to explore other deep eutectic solvent formulations with improved chemical stability. In addition, the high viscosity of these aluminum electrolytes is a very important factor affecting performance at increasing sulfur loadings. Therefore, further work is required for the development of less viscous electrolytes and advanced cathode structures with controlled porosity and low tortuosity to enable fast electrolyte ion transport.

## Results and Discussion

Increasing the amount of sulfur in Al–S batteries is crucial for achieving high energy from a light battery; therefore, increasing the sulfur content and loading in the cathode is essential. Herein we report a systematic study of the effect of sulfur loading in cathodes containing a high sulfur content of approximately 60 %. Increasing the sulfur loading was achieved by increasing the mass of the cathode loaded onto a Mo thin film substrate by increasing the wet thickness in the doctor blading coating process. This also resulted in an increase in the cathode thickness, which was measured by using a high‐precision thickness gauge.

**Figure 1:**
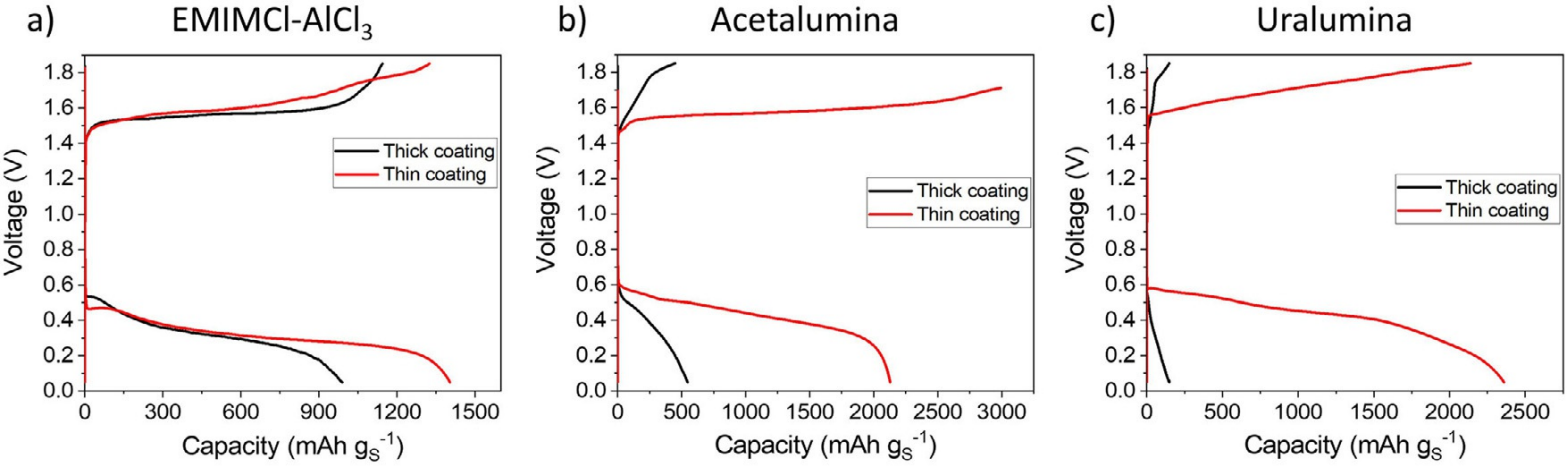
1st cycle voltage profiles of thick (139 μm) and thin (22 μm) cathode coatings using a) EMIMCl–AlCl_3_, b) acetalumina, and c) uralumina electrolytes. A specific current of 50 mA g_S_^−1^ and a 0.05–1.85 V voltage range was used. Cathodes contained 60 % sulfur and CNT as conductive additive.

The effect of increasing the cathode thickness on the electrochemical performance of Al–S batteries containing the three different electrolytes is shown in Figure 1. Low cathode loadings were prepared by using a thinner Mo foil substrate, which enabled an accurate evaluation of the cathode coating mass. This enables the reliable determination of the sulfur mass loading, which is required for normalizing the capacity values. Furthermore, reversible Al plating/stripping was achieved in all electrolytes (see cycling of Al–Al symmetrical cells in Figure S1 in the Supporting Information), in agreement with previous reports.16b, 16c, 17a, 18 The voltage window was selected to avoid the oxidation of the electrolyte at ≥2 V (Figure S2), in agreement with previous studies.^20^

As shown in Figure 1, increasing the sulfur loading dramatically affects the electrochemical response in the deep eutectic solvents acetalumina and uralumina, whereas for the ionic liquid EMIMCl–AlCl_3_, the effect of sulfur loading was much more moderate. This behavior can be understood in view of the viscosities of the different electrolytes (Table 2; see details of the calculations in Equation S3). The viscosity dramatically increased in the order EMIMCl–AlCl_3_<acetalumina<uralumina, and the capacity for thick cathode coatings decreased in the same way.

**Table 2:** Viscosity of electrolytes at room temperature (≈20 °C), as evaluated from the density values and the flow times.

Substance	Density [g mL^−1^]	Flow time [s]	Viscosity [MPa s]
EMIMCl–AlCl_3_	1.360	98	13
acetalumina	1.460	643	94
uralumina	1.564	1398	218

**Figure 2:**
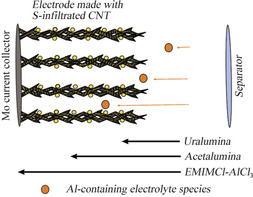
Limitations owing to slow transport of Al‐containing electrolyte species within the porous sulfur cathode in Al–S cells.

Consequently, it can be proposed that for the highly viscous electrolytes, mass transport limitations severely affect the performance and, consequently, only a small fraction of the sulfur present in the cathode participates in the electrochemical reactions, producing very small specific capacities. As shown in Figure 2, for slow transport of aluminum‐containing species within the electrolyte filling the cathode pores, most of the reaction take place in the cathode regions that are closer to the separator, and the regions closer to the current collector become under‐utilized. Because the reduction of sulfur produces Al_2_S_3_, which is an insulating solid, as the reaction proceeds, deposition of Al_2_S_3_ can block some of the pores of the sulfur composite cathode, thus slowing down mass transport and making some of the regions of the cathode poorly accessible. With faster mass transport, the reaction would be more homogeneous and the whole electrode could participate in the electrochemical reactions.

**Figure 3:**
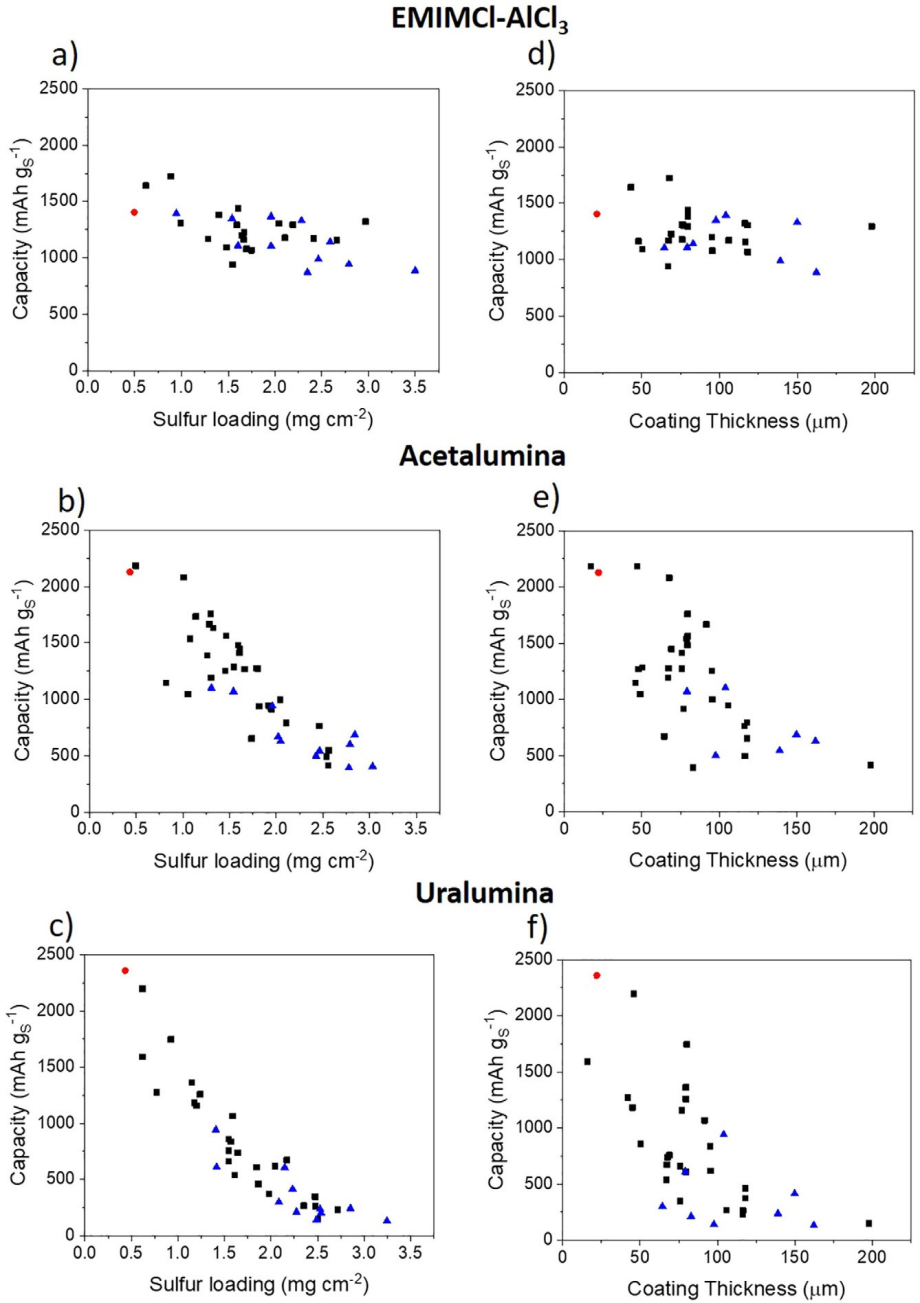
1st discharge capacity values plotted against the sulfur loading (a–c) or the cathode coating thickness (d–f) in EMIMCl–AlCl_3_ (a, d), acetalumina (b, e) and uralumina (c, f). The black square symbols show the results obtained with the electrodes coated on a thicker (25 μm) Mo foil, whereas the red circles show the results using a thinner (10 μm) Mo foil. In both cases, the electrodes contain 60 % sulfur and CNT as the only carbon source. The blue triangles show the results of electrodes made with a small amount (1–8 %) of other carbon additives mixed with CNT.

The plots of the experimental first discharge specific capacities, obtained for a variety of cathode thicknesses and cathode formulations, against the sulfur loading of the cathode (Figure 3 a–c) or the cathode thickness (Figure 3 d–f) are shown in Figure 3. The data gathered for these graphs is derived from 35–43 individual experiments per electrolyte. It is clear that for acetalumina, and especially for uralumina, a marked decrease in specific capacity was observed as the cathode was deposited as a thicker coating on the Mo thin film substrate. Conversely, in the ionic liquid EMIMCl–AlCl_3_, the effect of cathode thickness was much less marked. These findings provide further support to the hypothesis that the full utilization of thick electrodes in highly viscous electrolytes is difficult owing to mass transport limitations, which are exacerbated in Al–S batteries because the discharge product Al_2_S_3_ can clog the cathode pores. Interestingly, mass transport limitations have also been identified as a major issue limiting the capacity of state‐of‐the‐art Li–S cells.^21^

**Figure 4:**
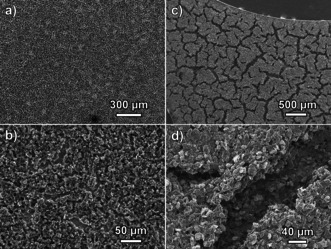
a, c) Low‐magnification and b, d) high‐magnification SEM images of sulfur electrodes with a thin coating (a, b; ≈45 μm cathode layer) and thick coating (c, d; ≈80 μm cathode layer), taken before cell assembly and cycling.

In summary, achieving high specific capacities with cathodes with a high sulfur loading requires the development of advanced cathode morphologies that are able to facilitate fast transport of Al‐containing electrolyte species through the whole porous cathode. For the cathodes developed here, the SEM images of thin and thick electrodes (Figure 4) show the presence of agglomerates and cracks. The thicker coating contains large cracks (Figure 4 c) that can also be observed directly by eye. Upon magnification, the SEM images show the presence of coating material at the bottom of the cracks (Figure 4 d), suggesting that the cracks were formed during drying of the ink deposit. This is in line with previous studies on the development of advanced cathodes for Li–S batteries, which showed that special combinations of different carbons and binders were required to produce thick sulfur cathode composites with good mechanical stability, no substantial cracking, and good electrochemical performance.^22^

**Figure 5:**
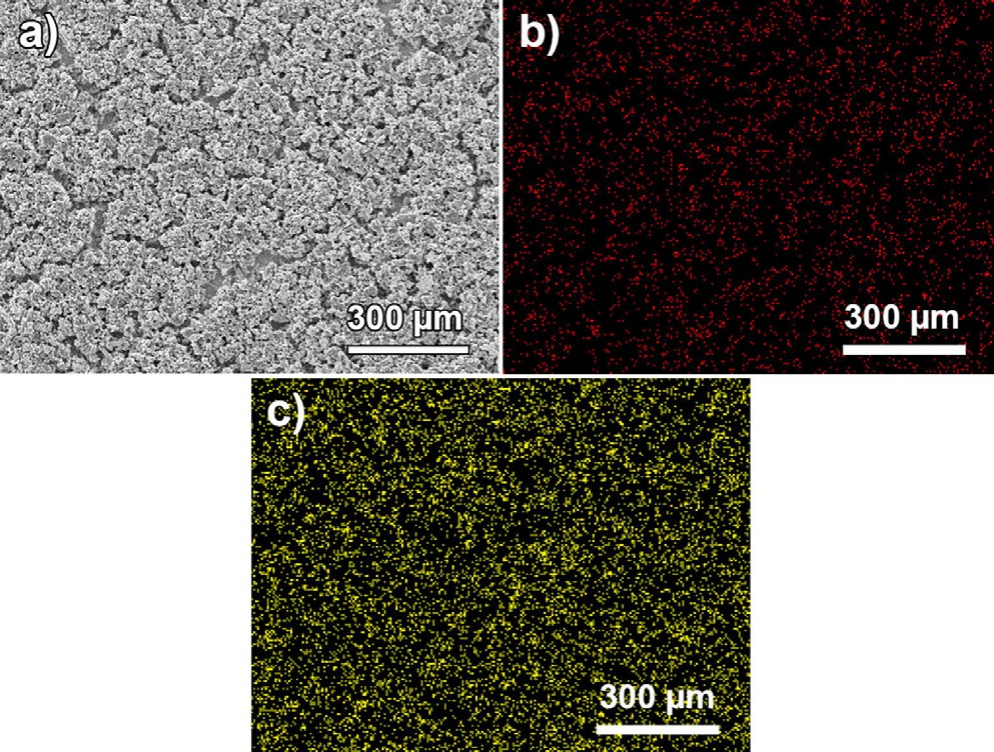
Energy‐dispersive X‐ray spectroscopy mappingof a sulfur cathode coating with a 60 μm thickness. SEM image (a) and associated distributions of carbon (b, red) and sulfur (c, yellow).

Elemental analysis of the cathodes by energy‐dispersive X‐ray spectroscopy (Figure 5) indicated a homogeneous distribution of sulfur and carbon, which could be attributed to the sulfur infiltration on the carbon nanotubes prior to the preparation of the electrodes.

**Figure 6:**
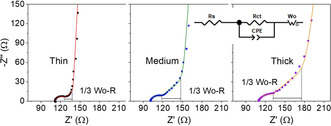
Nyquist plots of symmetrical cells containing thin (≈60 μm), medium (≈120 μm), and thick (≈190 μm) sulfur cathode coatings in uralumina. A 10 mV perturbation with a frequency range 200 kHz–10 mHz was used. The black segment shows the magnitude of one third of the total resistance associated with electrolyte ion transport through the porous electrode.

A quantitative analysis of electrolyte ion transport within the porous sulfur cathode was performed for the uralumina electrolyte with impedance measurements of the sulfur electrodes in symmetrical cells (Figure 6). These measurements were performed in a cell containing two nearly identical cathodes. Therefore, the impedance of the cell is simply two times the impedance of one cathode.^23^ In contrast, when impedance measurements are performed in Al–S cells, it is difficult to separate the contributions owing to the impedance of the aluminum anode and the sulfur cathode. In the Nyquist plots (Figure 6), a depressed semi‐circle that is caused by the contact resistance with the current collector is present and can be fitted to a resistor and a CPE element in parallel.23a, 24 This is followed by a straight line with an angle of 45°, reflecting the transport of the electrolyte ions through the porous electrodes, and then a straight, vertical line, reflecting the capacitive behavior of the electrode at low frequencies, at which only double‐layer charging of the carbon–electrolyte interphase takes place. Charge‐transfer reactions of the reduction of sulfur to polysulfides are absent in these measurements because the sulfur electrodes are at a high potential (≈1.3 V vs. Al).

As shown in Figure 6, the length of the 45° line (which measures the resistance associated with electrolyte ion transport within the porous electrode) increased as the electrode thickness increased. This supported the hypothesis that increasing the electrode thickness produces more severe mass transport limitations in highly viscous electrolytes. The results can be fitted to the equivalent circuit (Figure 6, inset), in which an open Warburg element is used to describe the transport of electrolyte ions through the pores of the electrode coupled to double‐layer charging at the carbon–electrolyte interphase.23a The analysis of the data is described in the Supporting Information (Equations S4 and S5) and the results of the analysis are summarized in Table 3. The analysis enables the evaluation of an effective tortuosity, which is a measure of the ratio between the ion transport path length and the geometrical thickness of the electrodes: higher tortuosity denotes a more intricate pathway and slower ion transport. Despite the high porosity of the electrodes employed in this study, the tortuosity towards ion transport is rather high, which can be attributed, at least in part, to the formation of dense agglomerates and cracks (Table 3). Further work will exploit the use of impedance measurements of sulfur composite electrodes in symmetrical cells to support the rational development of electrode/electrolyte formulations producing fast ion transport (that is, low Warburg resistance), and thus, the potential to deliver high specific capacities for electrode thicknesses relevant to commercial applications.

**Table 3:** Summary of the results of the analysis of the impedance measurements in Figure 6. The errors reported are obtained from the impedance fitting and propagation of experimental errors.

Electrode	*R*_s_ [Ω]	CPE‐T [F s^P−1^]	CPE‐P	*R*_ct_ [Ω]	Wo‐R [Ω]	Wo‐T [s]	Wo‐P	Porosity [%]	MacMullin number	Tortuosity
thin ≈60 μm	111.2±0.2	(6.5±2.1)×10^−5^	0.955±0.042	11.6±0.5	47.9±1.8	0.160±0.007	0.484±0.005	81±6	4.0±0.3	3.2±0.4
medium ≈120 μm	100.6±0.1	(8.4±1.9)×10^−5^	0.982±0.031	10.8±0.3	114.7±1.5	0.707±0.011	0.479±0.001	82±3	4.8±0.2	3.9±0.2
thick ≈190 μm	109.1±0.3	(1.02±0.45)×10^−4^	0.909±0.060	12.0±0.7	173.8±3.6	0.730±0.018	0.466±0.001	88±2	4.6±0.2	4.1±0.2

Coming back to the electrochemical results, close inspection of Figures 1 and 3 also showed that for acetalumina and uralumina, low cathode loadings produced very high specific capacity values, much higher than the theoretical capacity of 1672 mAh g^−1^ associated with the full reduction of sulfur to sulfide. Two previous studies of Al–S batteries found specific capacity values that were higher than the theoretical limit. The first was 1750 mAh g_s_^−1^ in an EMIMCl–AlCl_3_ electrolyte at 50 °C and the second was 2100 mAh g_s_^−1^ in an acetalumina electrolyte at room temperature.4a, 14 In both cases, it was argued that the high specific capacity values could be due to capacity contributions from carbon or side reactions, without providing any additional experimental evidence.

**Figure 7:**
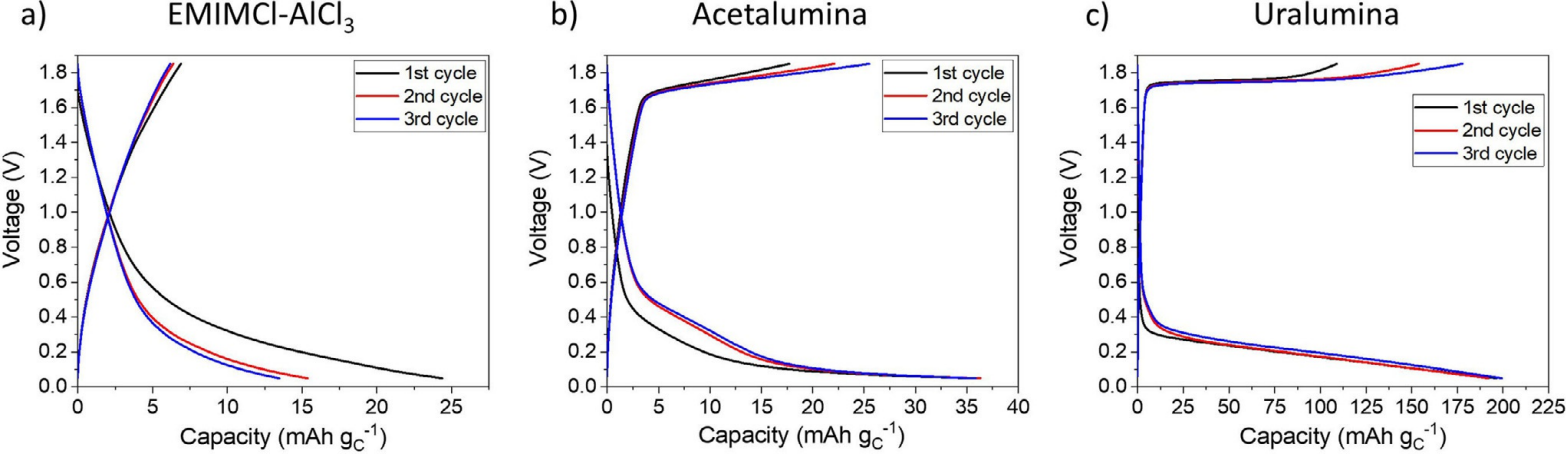
1st cycle voltage profile of cathodes made with only CNT and binder using a) EMIMCl–AlCl_3_, b) acetalumina and c) uralumina electrolytes. A specific current of 100 mA g_c_^−1^ and a voltage range of 0.05–1.85 V was used.

To evaluate the contribution of the carbon conductive additive to the specific capacity of the sulfur cathodes, additional experiments were performed with electrodes containing the carbon conductive additive and no sulfur. The voltage profile obtained with composite electrodes containing CNT and binder only (without any sulfur), when cycling against an aluminum counter electrode, for the three electrolytes under study are shown in Figure 7 (the specific capacities are normalized to the mass of carbon in the electrodes). The sulfur cathodes used in this work (Figures 1 and 3) had a ratio of carbon and sulfur mass content of 0.5 g_c_ g_s_^−1^ and, because the applied specific current (normalized to the mass of sulfur) was 50 mA g_s_^−1^, the value of the specific current normalized to the mass of carbon was 100 mA g_c_^−1^. For that reason, the electrodes in Figure 7 were cycled with a specific current of 100 mA g_c_^−1^. In addition, the electrodes were prepared with a loading of approximately 0.3 mg_c_ cm^−2^ to facilitate the full utilization of all the carbon present in the electrodes in the electrochemical reactions (for comparison, the carbon loadings in the sulfur cathodes used in Figures 1 and 3 were between 0.2 and 1.75 mg_c_ cm^−2^).

The results in Figure 7 clearly show that very small specific capacities were obtained with electrodes containing only carbon and binder. This demonstrated that electrochemical reactions taking place on the carbon conductive additive of sulfur cathodes produce a very small contribution to the total capacity. The same argument can be made by simply comparing the experimental capacities, without normalization to the mass of either sulfur or carbon. The capacities obtained with cells containing carbon‐only electrodes (Figure 7) were 12, 15, and 84 μAh in EMIMCl‐AlCl_3_, acetalumina, and uralumina electrolytes, respectively, whereas the capacities of the cells containing thin electrodes with sulfur (Figure 1) were 660, 876, and 971 μAh, respectively. This demonstrated that the contribution of the carbon response to the experimental specific capacities of the sulfur cathodes developed in this work is only minor.

Consequently, the reason that the experimental specific capacities were higher than the theoretical limit of 1672 mAh g_S_^−1^ can only be explained by the involvement of side reactions (most likely electrolyte degradation) in the discharge of the batteries. Although each sulfur atom can only receive a maximum of two electrons (which produces the theoretical capacity of 1672 mAh g_S_^−1^), additional processes can be induced during the discharge of the battery, which can consume more electrons, such as the reduction of urea or acetamide. Such degradation of the electrolyte is not observed in the electrodes without sulfur (Figure 7), which suggests that polysulfide species formed during the reduction of sulfur to sulfide are the triggers of the degradation reactions. Conversely, the ionic liquid EMIMCl–AlCl_3_ did not produce capacities that exceeded the theoretical limit, which suggests that this electrolyte was stable, at least under the experimental conditions employed here.

**Figure 8:**
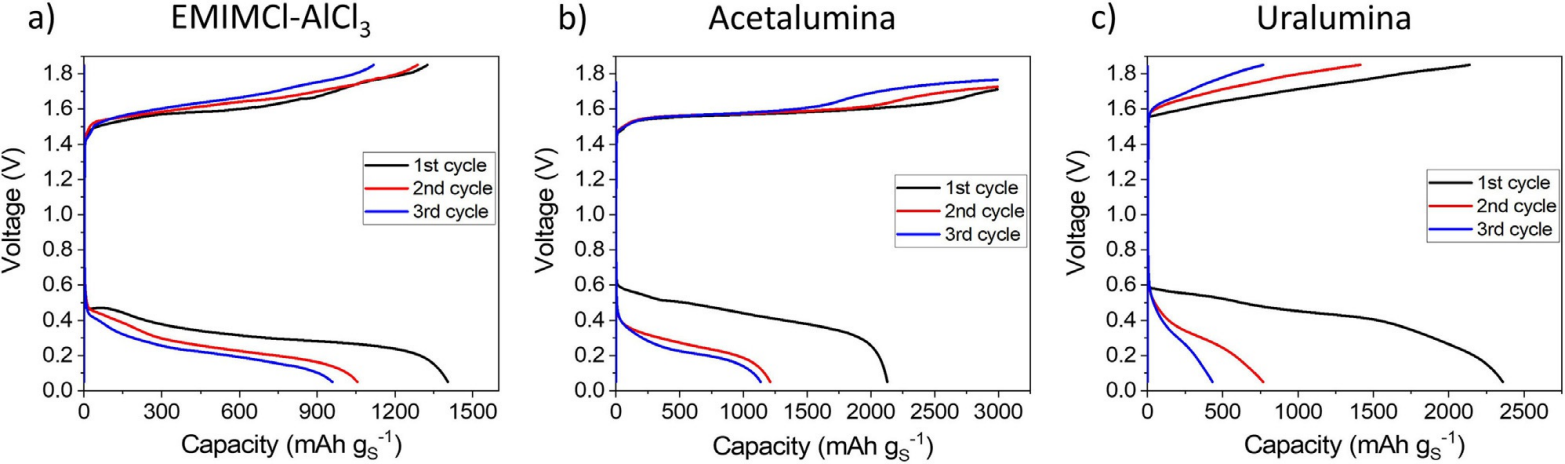
First three galvanostatic cycles of the thin cathode coatings shown in Figure 1.

**Figure 9:**
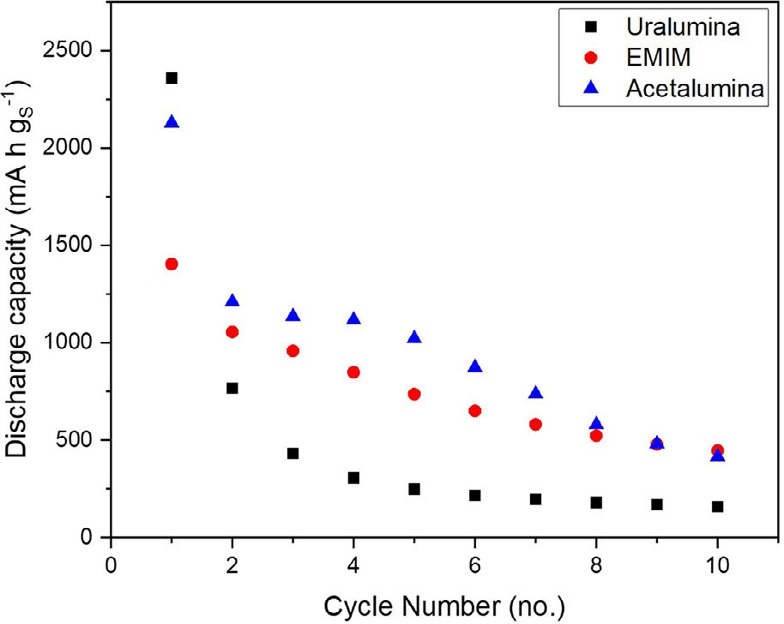
Discharge capacity versus cycle number data for the thin cathode coatings shown in Figure 1.

The evolution of the voltage profiles of Al–S cells during the first three cycles is shown in Figure 8. Perhaps as a result of the degradation reactions discussed above, the discharge voltage and discharge capacity in uralumina and acetalumina markedly decreased with cycling. The cycling stability in EMIMCl–AlCl_3_ was much better, but still major improvements are required to meet the requirements for commercial applications. The variation of the discharge capacity with cycling is shown in Figure 9, again demonstrating marked decreases with cycling in the deep eutectic electrolytes and better capacity retention in the ionic liquid EMIMCl–AlCl_3_.

**Figure 10:**
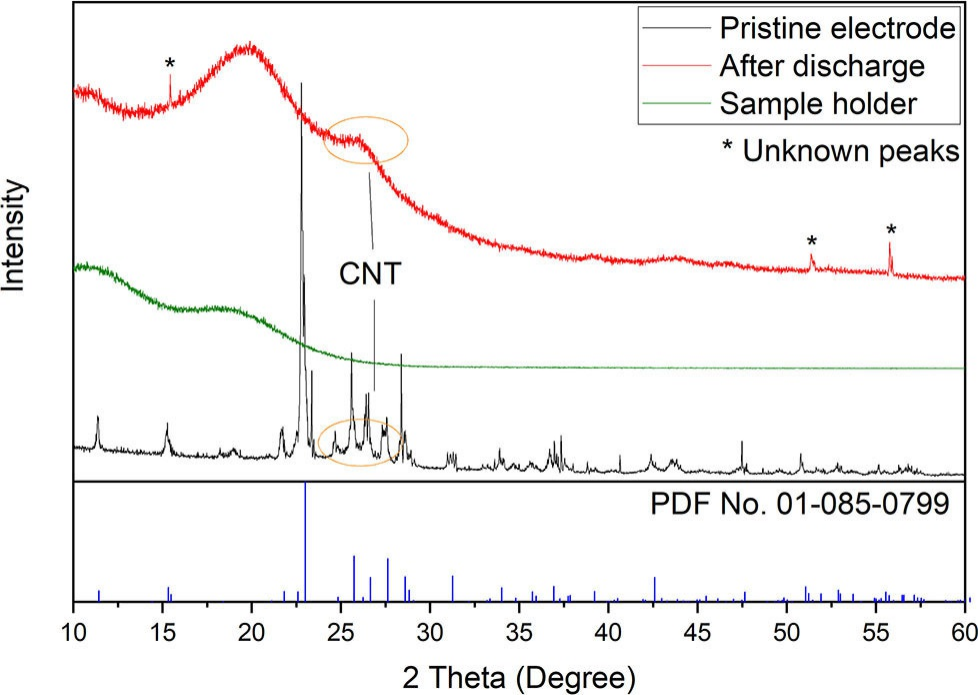
Powder XRD diffraction pattern (grazing incidence, incident angle of 5°) of a sulfur cathode with a thickness of 110 μm in the pristine state (black line) and after a full discharge to 0.05 V (red line). The diffraction pattern of crystalline sulfur (blue line) is also shown for comparison, as well as the diffraction pattern of the sample holder (green line).

Finally, X‐ray diffraction was used to characterize the cathodes before and after discharge in an Al–S cell (Figure 10). The diffraction pattern of the pristine electrode clearly showed the presence of crystalline sulfur. Then, the electrode was discharged in an EMIMCl–AlCl_3_ electrolyte, after which the battery was disassembled inside an argon glovebox and the discharged cathode was placed in an air‐tight XRD sample holder. All the peaks associated to crystalline sulfur disappeared in the diffraction pattern of the discharged electrode, indicating that all the sulfur was consumed in the electrochemical discharge reactions. Only two broad peaks, which were attributed to the sample holder, and three very small unidentified peaks were present in the XRD pattern. Therefore, the XRD data suggest that the discharge reaction consumed all the sulfur and produced no crystalline degradation products. Unfortunately, the formation of crystalline Al_2_S_3_ was also not observed, which could be because Al_2_S_3_ was formed in amorphous form or because the amount of crystalline Al_2_S_3_ formed in the present experiments was below the limit of detection. Previous studies of Al–S batteries have also been unable to identify the formation of crystalline Al_2_S_3_ by XRD of the discharged electrodes.4a, 8 The high reactivity of Al_2_S_3_ and the electrolyte with trace amounts of water also makes the characterization of the discharged electrode particularly challenging and requires special transfer systems with stringent water‐free atmospheric conditions.^25^

## Conclusions

A systematic study of the effect of the thickness of the sulfur cathode on the electrochemical response of Al–S batteries is presented for three relevant electrolytes: the most popular EMIMCl–AlCl_3_ and two deep eutectic electrolytes made by mixing either acetamide or urea with AlCl_3_. For the deep eutectic electrolytes, a very dramatic decrease in capacity with increasing sulfur cathode thickness was observed, which was attributed to the high viscosity of these electrolytes that hampers full sulfur utilization owing to slow electrolyte ion transport within the porous electrode. SEM characterization of the cathodes showed that their morphology was not ideal, with big cracks and agglomeration of particles on the thicker cathodes, which explained the slow electrolyte ion transport through those packed agglomerates. Impedance measurements in symmetrical cells demonstrated that the resistance associated with electrolyte ion transport markedly increases as the cathodes thickness increased, and analysis of the results revealed that the cathodes had high effective tortuosity for electrolyte ion transport, supporting the hypothesis that slow electrolyte ion transport limits the capacity achievable with these cathode structures. The electrochemical results of the Al–S cells also showed that very high capacities were obtained with deep eutectic electrolytes with very thin sulfur cathodes, with specific capacities exceeding the theoretical limit of 1672 mAh g_S_^−1^ expected for full reduction of sulfur to sulfide. An analysis of the electrochemical response obtained with carbon electrodes with no sulfur showed that the contribution of the capacity owing to carbon was only minor. Therefore, the high specific capacities were explained by the occurrence of side reactions (electrolyte degradation) that took place alongside the sulfur electrochemical reactions. However, XRD characterization of the pristine and discharged electrode in EMIMCl–AlCl_3_ suggested that the discharge reaction consumed all the sulfur and produced no crystalline degradation products.

In summary, this work demonstrates that the systematic study of the performance of Al–S cell as a function of the sulfur cathode thickness is a very powerful approach to identify and quantify issues with electrolyte degradation or limitations owing to mass transport, and the approach can be easily applied to study new cathode and electrolyte formulations beyond the state of the art of the Al–S battery system.

## Experimental Section

### Cathode preparation

Sulfur (Aldrich) and multiwalled carbon nanotubes (CNTs, 98 % basis, Sigma–Aldrich) were mixed (sulfur/carbon ratio=2:1 *w*/*w*) by pestle and mortar before being transferred into a 23 mL polytetrafluoroethylene (PTFE)‐lined autoclave (Parr Instrument Company). The autoclave was then placed into a preheated oven (Genlab Ltd. MINO/6, 155 °C, 6.5 h) to allow sulfur impregnation to occur. After cooling down to room temperature, a black powder was collected. Polyethylene oxide (PEO, *M*_w_=600 000, Sigma–Aldrich) and polyvinylpyrrolidone (PVP, *M*_w_=40 000, Aldrich) were used as binder (PEO/PVP ratio of 2:1 *w*/*w*, ≈12 wt % overall within the coating) and dissolved in a 2:1 *v*/*v* mixture of acetonitrile (HiPerSolv, ACS) and ethanol (Fisher, ≥99.8 % analytical grade) under a fume hood. The PEO/PVP combination has been used before in Li–S batteries and was employed here because it provides good dispersion and mechanical stability.22a, 26 The sulfur‐impregnated CNTs (≈270 mg) were added into the binder solution and stirred with a stirring bar for either 2 h or 3 days. A precut piece of Mo foil (25 μm thickness, ≥99.9 % trace metals basis, Sigma–Aldrich) was cleaned by using ethanol before it was coated with ink using a doctor‐blade coater. After the acetonitrile and ethanol evaporated, the coated electrode sheet was cut into round discs (Ø=11 mm, Hohsen hand‐held precision punch). The discs were placed into a Büchi tube and dried at room temperature (typically, 20–25 °C), under vacuum for no less than 2 days. Each electrode was weighed (OHAUS Adventurer^TM^, AR0640) inside an Ar‐filled glovebox (H_2_O≤4.0 ppm, O_2_≤0.1 ppm) before use, to obtain the precise weight of the active material. The standard electrode composition was 58.8:29.4:7.9:3.9 (S/CNT/PEO/PVP, wt %) for simplicity, this is referred to as 60 % sulfur. A standard coating using the procedure above required 1.4 mL acetonitrile and 0.7 mL ethanol and, when combined with a 300 μm doctor blade setting, produced a coating thickness of approximately 65 μm.

The cathode coating thickness was systematically varied in two ways: (i) by using different settings for the wet thickness on the doctor blade coating apparatus to change the amount of ink coated, or (ii) by varying the solvent quantity in the ink thereby producing thinner/thicker coatings. Additional experiments were performed with electrodes with very small mass loading (that is, very thin cathode coatings). To reliably determine the cathode mass loading in these electrodes (and, thus, the sulfur loading), the coatings were made on thinner Mo foils (10 μm, 99.9 % purity, Sigma–Aldrich). The Mo foils were punched into 11 mm‐diameter discs, which were pre‐weighed before coating and then after coating and drying. The thinner Mo discs typically weighed between 9.9–10.1 mg whereas the cathode loading mass of the thin coatings was approximately 0.7–0.8 mg. The balance error was ±0.05 mg, which produced an error in the mass of the coating of ±0.07 mg; therefore, the error in the reported specific capacities is ≤10 %.

Additional results from other experimental electrodes are also reported and are labeled as “containing other carbon additives”. The additives used were TIMCAL C65, graphene nanoplates (Ossila Ltd.), and acetylene black (50 % compressed, Shawinigan Black, Chevron Phillips Chemical Company LP.) and typically account for 1–8 wt %. Among the results of electrodes with additives, in a few cases the electrodes contained 50 wt % rather than 60 wt % sulfur.

The thicknesses of the cathode coatings on the Mo foil were measured with an ABS Digital Thickness Gauge (resolution: 1 μm). All electrodes were numbered and packaged separately.

CHNS elemental analysis, performed by MEDAC Ltd., demonstrated that there had been no sulfur loss during the impregnation step, a 2:1 sulfur/carbon ratio was observed after the impregnation (Table S1). Additionally, no sulfur loss was detected during the drying of the electrodes in a Büchi line at room temperature, and no significant change in the mass of the electrodes before and after drying was observed. Additionally, TEM images (Hitachi 7700 TEM at 100 kV, Figure S3) of the carbon nanotubes showed that the impregnation process does not deform the structure.

### Electrolyte preparation

1‐Ethyl‐3‐methylimidazolium chloride–aluminum chloride (EMIMCl–AlCl_3_; EMIMCl/AlCl_3_ molar ratio=1:1.5) was purchased from Sigma–Aldrich. Acetalumina (acetamide/AlCl_3_ molar ratio=1:1.5) and uralumina (urea/AlCl_3_ molar ratio=1:1.5) were generously provided by Dr. Christopher Zaleski (Scionix Ltd.), Dr. Igor Efimov, and Prof. Karl Ryder (University of Leicester). The electrolytes were used as received.

### Cell construction

The electrochemical cells were made of materials that are resistant against corrosion in contact with the electrolytes. Preliminary experiments were conducted with Swagelok cells made of PTFE, and it was later found that Swagelok cells made of aluminum produced the same results (Figure S5). Although aluminum corrodes at high potentials, it is possible to use the cell body made of aluminum since the cell body is electrically disconnected from both electrodes (anode and cathode), and therefore, it remains at the open circuit potential at which aluminum is stable. The cell was sealed with perfluoroalkoxy alkane (PFA) ferrules, and Mylar film was used to line the inside of the Al cell body, preventing contact between the electrodes and the cell body. Sulfur electrodes (11 mm diameter discs) were used as cathodes, high purity Al discs (0.2 mm thick, Puratronic, 99.997 % metals basis, 11 mm diameter discs) were used as anodes. Two Whatman GF/F glass microfiber filters (12 mm diameter discs) were used as separators and 120 μL of electrolyte were added to the cell. Solvent Safe^TM^ 200 pipet tips were used owing to their increased resistance to acidic environments compared with conventional pipette tips. An aluminum bar (purity ≥97.5 %) was used as the current collector for the Al anode and a tungsten bar (purity ≥99 %) was used as current collector for the cathode.

Prior to cell assembly, all the cell components were washed in ethanol (Fisher, >=99.8 % analytical grade), sonicated (Cole–Parmer, 08891‐26) for 15 min and dried in an oven (Genlab E3, E3DWC100/N) at 80 °C. Prior to drying, the cells were partially assembled and checked for short‐circuiting with a multimeter. After drying, all the components were transferred into an Ar‐filled glovebox (H_2_O≤4.0 ppm, O_2_≤0.1 ppm). The electrodes were directly transferred to the glovebox inside a Büchi tube to avoid air exposure. Cells assembly was performed inside the glovebox.

### Electrochemical testing and characterization

After assembly inside the glovebox, the cells were placed in a Memmert climatic chamber set to 25 °C for the electrochemical characterization. The cells were allowed to equilibrate for 6 h and then galvanostatic cycling with potential limitation (GCPL) experiments were performed by applying a lower and upper voltage limit of 0.05 V and 1.85 V, respectively, using a VMP3 potentiostat (BioLogic Science Instruments). The applied specific current (normalized to the mass of sulfur) was 50 mA g_S_^−1^. Discharge to 0.05 V was performed first, followed by charging to 1.85 V, and the cells were cycled for 10 cycles. Potentiostatic impedance spectroscopy (PEIS) measurements were performed in a symmetrical cell configuration (i.e., using two sulfur electrodes with almost identical weight and tungsten bars as current collectors, see Figure S4). The PEIS measurements were performed with a voltage amplitude of 10 mV and frequencies of 200 kHz–10 mHz. The XRD patterns were recorded at room temperature with a Rigaku Smartlab diffractometer (Rigaku Corporation) using Cu_Kα_ radiation operated at 45 kV, 150 mA. Samples were placed in a special X‐ray transparent dome‐shaped sample holder for air‐sensitive materials purchased from Bruker (Ø=55.5 mm PMMA disc with Ø=25 mm×1 mm specimen well). A piece of glass was used as a flat surface to elevate the electrode during measurements. The morphology and element distribution of raw materials and sulfur electrodes was studied using a JEOL JSM59 scanning electron microscope (15 kV) with an Oxford instruments energy‐dispersive X‐ray spectroscopy (EDS) attachment. Viscosity measurements were taken using a Cannon–Fenske viscometer tube (Sigma–Aldrich) and the experiment was recorded using a mobile phone camera (Samsung Galaxy S10+). The viscosity values were calculated from the time required for the electrolytes to flow between two marks in the viscometer, as estimated from the video.

## Conflict of interest

*The authors declare no conflict of interest*.

## Supporting information

As a service to our authors and readers, this journal provides supporting information supplied by the authors. Such materials are peer reviewed and may be re‐organized for online delivery, but are not copy‐edited or typeset. Technical support issues arising from supporting information (other than missing files) should be addressed to the authors.

Supplementary
