# Supplementary material for: A Critical Evaluation of the Effect of Electrode Thickness and Side Reactions on Electrolytes for Aluminum–Sulfur Batteries
Source: ChemSusChem. 2020 May 15;13(13):3514–23. doi: 10.1002/cssc.202000447 (PMC7384068; doi:10.1002/cssc.202000447)
Supplement: Supplementary file 1 — Supplementary [file CSSC-13-3514-s001.pdf]

ChemSusChem

Supporting Information

A Critical Evaluation of the Effect of Electrode Thickness and Side Reactions on Electrolytes for Aluminum–Sulfur Batteries

John Lampkin+,[a] He Li+,[a] Liam Furness,[a] Rinaldo Raccichini,[a, b] and Nuria Garcia-Araez*[a]

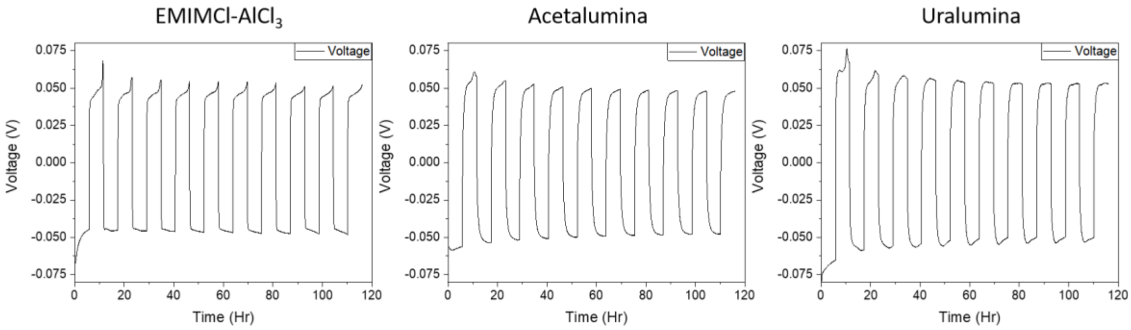

Figure S1. Cycling of Al-Al symmetrical cell in 1-Ethyl-3-methylimidazolium chloride-aluminium chloride (EMIMCl-$AlCl_3$, 1:1.5 molar ratio), Acetalumina (acetamide: $AlCl_3$ = 1:1.5 molar ratio) and Uralumina (urea: $AlCl_3$ = 1:1.5 molar ratio) at a current density of 67.3 µA/cm² (10 cycles, total measurement time: 117 h).

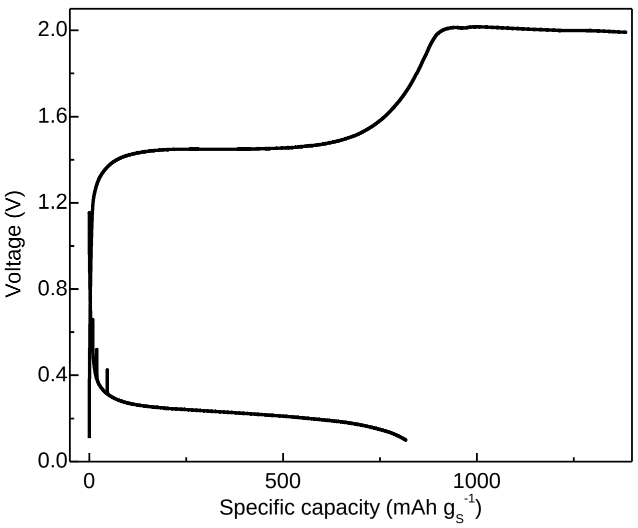

Figure S2. 1st cycle voltage profile of an Al-S battery with an extended upper voltage limit, showing a voltage plateau at ca. 2 V associated to the oxidation of the electrolyte.[1] Electrolyte: EMIMCl-$AlCl_3$, 1:1.5 molar ratio. Sulfur composite electrode composition: sulfur (24.4%), acetylene black (56.6%) and PTFE (10%). Specific current: 50 mA g_S^{-1}.

Table S1. CHN + S analysis performed by MEDAC Ltd. on Sulfur impregnated nanotubes with a ratio of 2:1.

ELEMENT	C	H	N	S					
% Theory									
% Found 1	32.74	<0.10	<0.10	67.66					
% Found 2	32.93	<0.10	<0.10	67.26					

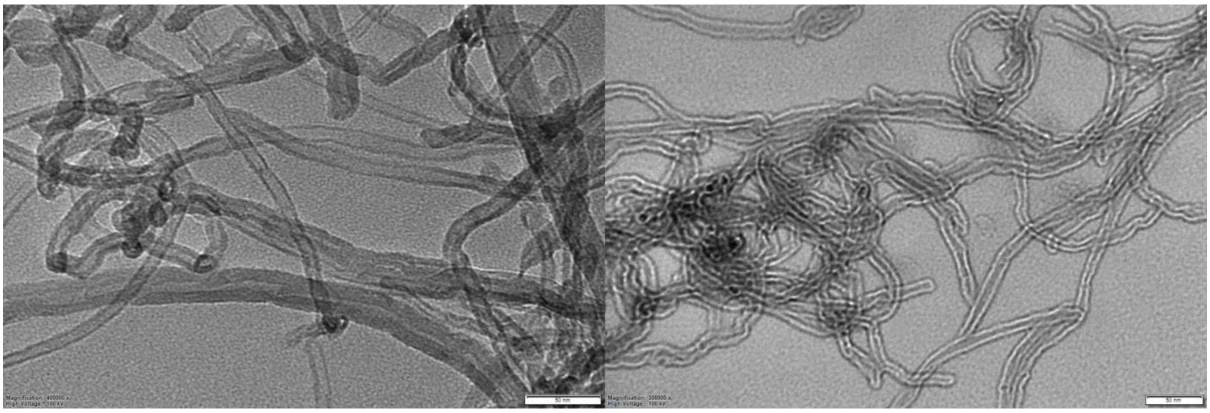

Figure S3. TEM images of CNT98 (left) and CNT98 impregnated with sulfur (right). Experiments were conducted using a Hitachi 7700 TEM at 100 kV.

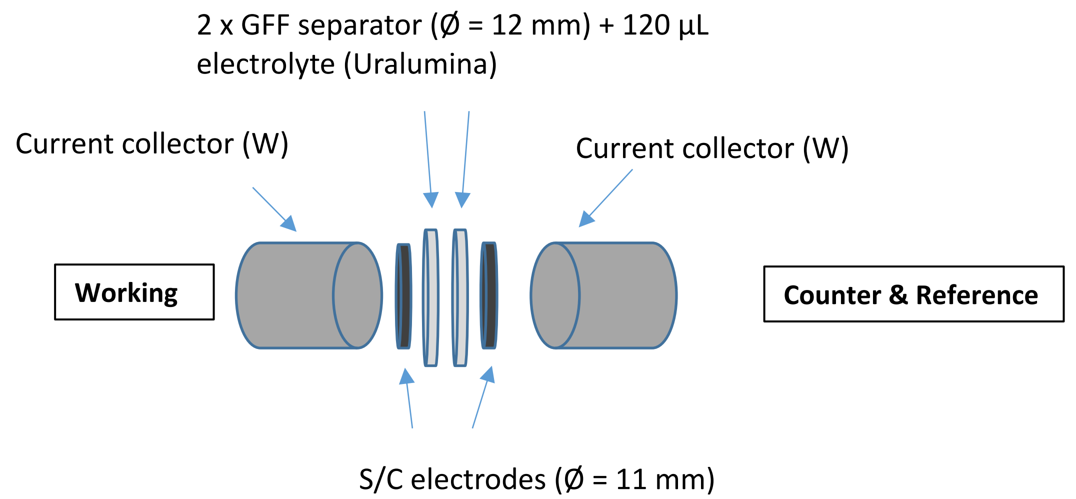

Figure S4. Illustration of a symmetrical Al Swagelok cell used for impedance measurements using 60% sulfur electrodes.

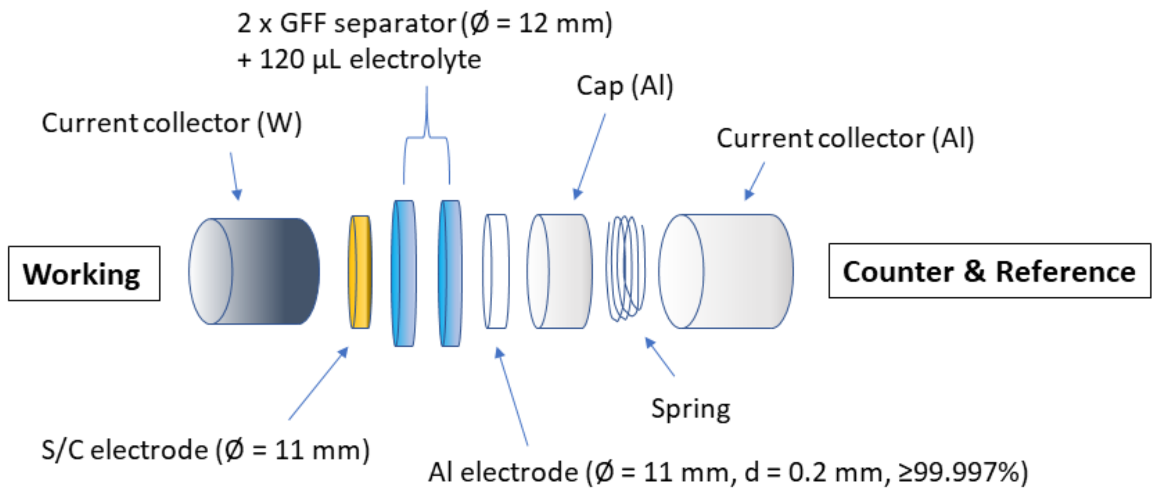

Figure S5. Illustration of Al Swagelok cell.

Equation S1. The average discharge voltage, V_{DISCH}, was calculated using the following equation:

$$V_{DISCH} = \frac{\int_0^{Q_{FINAL}} VdQ}{Q_{FINAL}}$$

where:

V: discharge voltage

Q = specific capacity

Q_{FINAL} = total specific capacity at the cut off voltage

Equation S2. The specific energy of the initial discharge (normalized to the total mass of the sulfur and aluminium electrodes) was calculated from:

Specific energy of initial discharge = $V_{DISCH} * Q_{FINAL}/(100/\%wt. + 0.561)$

where:

V_{DISCH} = average discharge voltage

Q_{FINAL} = total specific capacity at the cut off voltage

$\%wt.$ = mass % of sulfur in the sulfur electrode

This calculation includes the mass of the aluminium electrode required to react with the sulfur electrode to produce Al_2S_3. This calculation takes into account that each gram of sulfur in the sulfur electrode requires a mass of 0.561 g of aluminium for the formation of Al_2S_3, and that each gram of sulfur in the sulfur electrode is associated to a total mass of the sulfur electrode of 100/%wt., where %wt. is the mass percentage of sulfur in the sulfur electrode.

Equation S3. The dynamic viscosity of the electrolyte, η, was calculated from:

$$\eta = K * t * \rho$$

where K is the viscometer constant (K= 0.1 cSt/s), t is the time taken for the electrolyte to freely flow between two marks in the viscometer and ρ is density of the electrolyte.

Equation S4. The porosity of electrodes is calculated as follows:

$$V_{bulk} = \frac{mass\ of\ coating}{bulk\ density\ of\ materials}$$

$$V_{total} = \pi r^2 h$$

$$Porosity = \varepsilon = \frac{V_{total} - V_{bulk}}{V_{total}}$$

where

V_{bulk} is the volume that the electrode would occupy if it had no pores

V_{total} is the total, experimental volume of the electrode, obtained by using the experimental radius, *r*, and thickness, *h*, of the electrode. Note that in these calculations, the thickness of the electrode does not include the Mo foil substrate.

The calculations were done considering that the density of the cathode materials is 2 g cm^3.

Equation S5. The calculations of the MacMullin number, N_M, and tortuosity, τ, are described below.

$$k_{ELECTRODE} = \frac{h}{R_{ELECTRODE}\, \pi\, r^2}$$

$$N_M = \frac{k_{BULK}}{k_{ELECTRODE}}$$

$$\tau = N_M\, \varepsilon$$

where:

$k_{ELECTRODE}$ is the conductivity of the electrolyte inside the porous electrode

$R_{ELECTRODE}$ is the Warburg resistance of the electrode (obtained from fitting impedance data: the quantity Wo-R reported in table 3 in the main article)

h is the thickness of the cathode coating (without the Mo foil)

r is the radius of the electrode

k_{bulk} is the bulk conductivity of the electrolyte. For Uralumina, $k_{bulk} = 5.3\times10^{-4}$ S cm^{-1}, as evaluated by Dr. Christopher Zaleski (Scionix Ltd.), Dr. Igor Efimov and Prof. Karl Ryder (University of Leicester).

N_M is the MacMullin number,

ε is the electrode porosity (obtained using equation S4)

τ is the electrode tortuosity

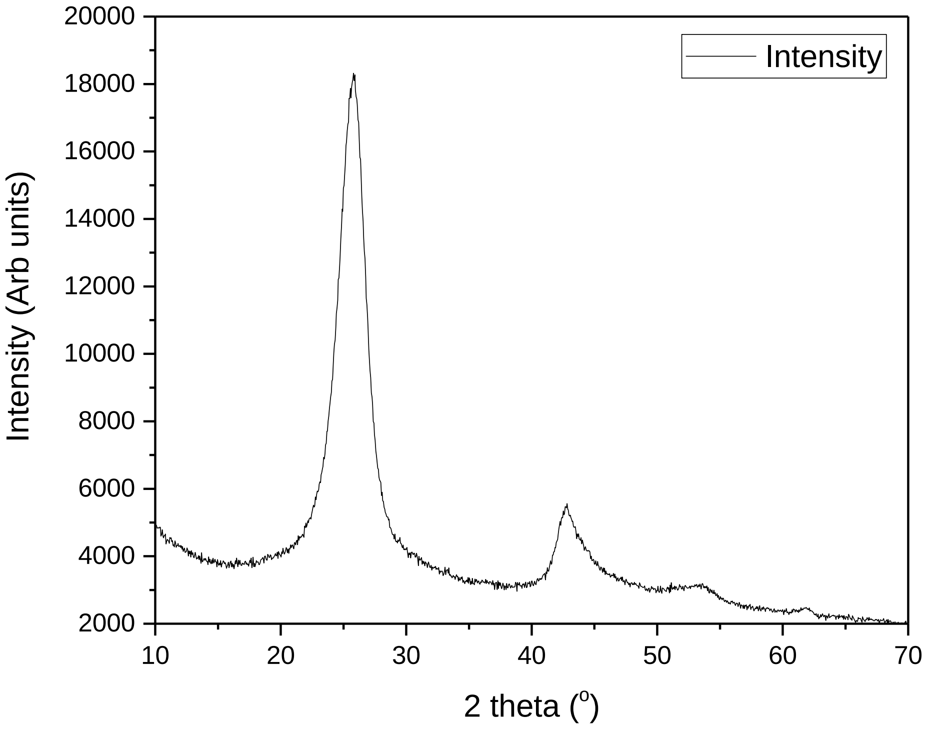

Figure S6. Powder X-ray diffraction pattern of the carbon nanotube (98 % carbon basis), CNT98. The pattern was recorded with Bruker D2 phaser (Bruker Corporation) with Cu K_{α} radiation operated at 30 kV, 10 mA between ($10<2\theta/°<70$).

References

[1] a) M. Lipsztajn, *Journal of The Electrochemical Society* **1983**, *130*, 1968; b) M. Lipsztajn, R. A. Osteryoung, *Inorganic Chemistry* **1984**, *23*, 1735-1739; c) Z. J. Karpinski, R. A. Osteryoung, *Inorganic Chemistry* **1985**, *24*, 2259-2264.
